# Serial Myocardial Imaging after a Single Dose of Thallium-201

**Published:** 2014

**Authors:** Takahiko Kamata, Tatsuya Kawasaki, Tadaaki Kamitani, Hiroki Sugihara

**Affiliations:** 1 Department of Cardiology, Matsushita Memorial Hospital, Osaka, Japan

**Keywords:** Angioplasty, Kinetics, Myocardial ischemia, Thallium

## Abstract

Although thallium-201 exercise scintigraphy has been established for the detection of myocardial ischemia and viability, little is known regarding the myocardial thallium-201 kinetics during angioplasty. Herein, we report a 77-year-old man with angina pectoris, in whom serial myocardial imaging after a single dose of thallium-201 was helpful in identifying not only the culprit lesion and myocardial viability, but also the dynamic changes in myocardial perfusion during angioplasty. Thallium-201 images after exercise showed a perfusion defect in the inferior wall, with a trivial redistribution 3 hours after the exercise and a marked improvement 24 hours later. Coronary angiography, performed 27 hours after exercise scintigraphy, showed severe stenosis in the right coronary artery. Guidewire crossing of the lesion interrupted the antegrade flow, which was restored after balloon dilation and stent implantation. Thallium-201 images, 2 hours after angioplasty (i.e., 30 hours after exercise), showed a decreased tracer uptake in the inferior wall, which improved the next day (i.e., 48 hours after exercise). Cardiac biomarkers were negative in the clinical course.

## Introduction

Although thallium-201 exercise scinti-graphy has been established for detecting myocardial ischemia and viability (1), little is known regarding the kinetics of thallium-201 during angioplasty. Herein, we report a patient with angina pectoris, in whom serial myocardial imaging after a single dose of thallium-201 was helpful in identifying not only the culprit lesion and myocardial viability, but also the dynamic changes in myocardial perfusion during angioplasty.

## Case report

A 77-year-old man was referred to our hospital with a 1-month history of exertional chest pain. The patient reported that the chest pain had often occurred several minutes after he started to walk. The pain gradually spread to the shoulders and jaw and lasted for a few minutes after exercise cessation. The patient also had a previous history of hypertension and dyslipidemia. He occasionally consumed alcoholic drinks and did not smoke.

The medications included amlodipine (5 mg daily), carvedilol (10 mg daily), temocapril (2 mg daily), aspirin (100 mg daily), and lansoprazole (15 mg daily).

The patient's vital signs and physical examination results were unremarkable. The results of electrocardiogram, chest radiograph, and echocardiogram were normal, similar to routine blood examination results. Coronary computed tomography angiography showed multiple stenotic lesions, which were moderate to severe in severity. To determine the culprit lesion, maximal symptom-limited exercise scintigraphy with thallium-201 was scheduled.

The exercise began at a workload of 25 watts, which increased by 25 watts every 2 minutes, using a bicycle ergometer under continuous monitoring with the Mason-Likar lead system. Exercise was discontinued due to chest pain with horizontal ST-segment depression of 1 mm in inferior leads. The maximal workload, heart rate, and rate-pressure product were 50 watts, 83 bpm, and 13,944, respectively.

Thallium-201 (111 MBq or 3 mCi) was injected intravenously at the peak of exercise. A total of 36 images over a 180-degree anterior arc were acquired 5 minutes or 3 hours after tracer injections with a digital gamma camera, equipped with a low-energy, high-resolution, and parallel-hole collimator. The acquisition lasted 50 beats per projection, stored in a matrix of 64×64 pixels, and the images were reconstructed using a Hanning filter without attenuation or scatter correction.

**Figure 1:**
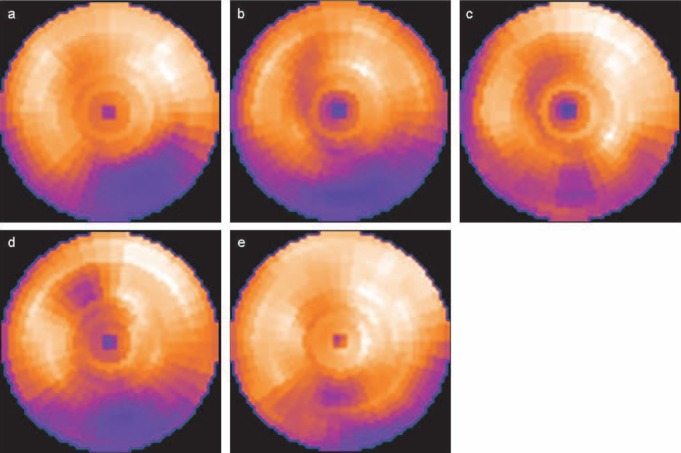
Thallium-201 bull's-eye map after exercise shows a perfusion defect in the inferior wall (A). A trivial redistribution is observed 3 hours after exercise (B). The bull's-eye map 24 hours after exercise shows a marked improvement in the inferior wall (C). A notably decreased tracer uptake is seen in the inferior wall 2 hours after angioplasty (i.e., 30 hours after exercise) (D). The inferior uptake is improved 20 hours after angioplasty (i.e., 48 hours after exercise) (E).

The bull's-eye map after exercise showed a perfusion defect in the inferior wall (Figure 1a). The redistribution of thallium-201 was trivial 3 hours after the exercise (Figure 1b), although left ventricular asynergy was not detected in echocardiography. Additional scintigraphic imaging was performed 24 hours after the exercise, showing a marked improvement in the inferior wall (Figure 1c).

**Figure 2:**
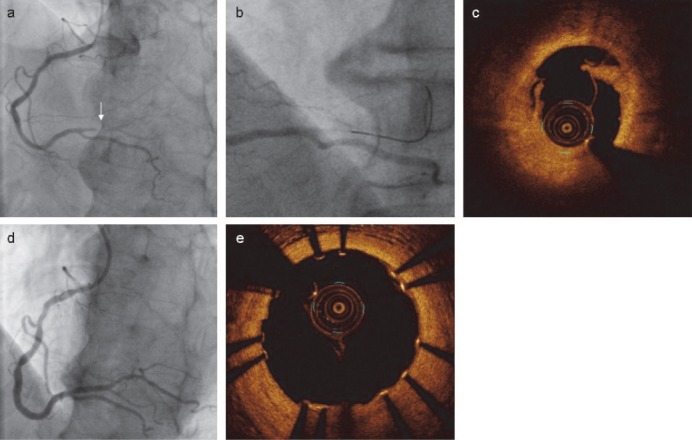
Right coronary angiography shows the severe stenosis of the posterolateral branch with antegrade flow (A, arrow). The antegrade flow disappears after guidewire crossing of the lesion (B). Optical coherence tomography after balloon angioplasty shows coronary dissection (C). Stent implantation was successfully performed with no residual stenosis (D). No dissection is detected on optical coherence tomography (E).

Coronary angiography, performed 27 hours after exercise scintigraphy, showed mild-to-moderate stenosis of the mid-portion of the left anterior descending artery and the left circumflex artery, and also severe stenosis of the posterolateral branch of the right coronary artery (Figure 2a, arrow; Video S1). Guidewire crossing of the lesion interrupted the antegrade flow (Figure 2b; Video S2), which was restored immediately after balloon dilation within a few minutes.

Coronary dissection, detected on optical coherence tomography (Figure 2c), was successfully treated by stenting (Figures 2d and 2e; Video S3). The bull's-eye map, with an acquisition time of 60 beats per projection, which was reconstructed 2 hours after angioplasty (i.e., 30 hours after exercise with thallium-201), showed a decreased tracer uptake in the inferior wall (Figure 1d), with an improvement within the next day (i.e., 48 hours after the exercise) to the same level as in the pre-angioplasty thallium-201 images (Figure 1e).

Cardiac biomarkers were negative during the clinical course. The day after angioplasty, the levels of high-sensitivity cardiac troponin T and creatine kinase MB isoenzyme were 0.007 ng/ml (reference range <0.014 ng/ml) and 7 IU/l (reference range <25 IU/l), respectively. The patient has been in a stable condition without any chest symptoms for more than 6 months.

## Discussion

The initial distribution of thallium-201 in myocardium is determined by regional myocardial blood flow and tracer content; the determinant of thallium-201 redistribution is a balance between the influx (i.e., continued myocardial extraction from systemic circulation) and efflux (i.e., the intrinsic myocardial washout) (2-4).

In an experimental study on anesthetized dogs (2), the extraction fraction of thallium-201 was almost constant over a wide range of coronary perfusion pressure, whereas the intrinsic washout rate of thallium-201 was markedly prolonged in association with decreased coronary perfusion pressure. These findings may indicate that the efflux, compared to influx, is a major determinant of thallium-201 myocardial redistribution except for patients with a very low coronary perfusion pressure, such as those with acute myocardial infarction. Given the rapid recovery of thallium-201 washout rate after the improvement of coronary perfusion pressure (2), angioplasty might affect the serial images of thallium-201 if administered before such invasive procedures.

In our case, changes in thallium-201 uptake in the inferior wall after exercise (3 and 24 hours after exercise) indicated that the culprit lesion was at the posterolateral branch of the right coronary artery, with myocardial viability in the inferior wall (1). Interestingly, thallium-201 images, acquired 30 and 48 hours after tracer injections, were acceptable in quality and were indicative of a transient decrease of tracer uptake in the inferior wall after angioplasty.

Although the exact involved mechanism remains unclear, we may safely consider that it was not the result of distal embolism, but increased thallium-201 washout rate after reperfusion (2, 5), possibly accompanied by hyperemia due to the right coronary dilation, following transient artery occlusion during angioplasty (6). It is not surprising that the redistribution of thallium-201 was observed in the inferior wall on the following day, owing to the disappearance of an increased washout rate after angioplasty. This speculation is, in part, supported by the lack of a slow flow after balloon angioplasty or stenting and lack of elevated cardiac biomarkers in the clinical course.

Our case highlights that serial imaging after a single dose of thallium-201 may be helpful in identifying not only the culprit lesion and myocardial viability, but also the dynamic changes in myocardial perfusion during angioplasty.
